# The association of remotely-sensed outdoor temperature with blood pressure levels in REGARDS: a cross-sectional study of a large, national cohort of African-American and white participants

**DOI:** 10.1186/1476-069X-10-7

**Published:** 2011-01-19

**Authors:** Shia T Kent, George Howard, William L Crosson, Ronald J Prineas, Leslie A McClure

**Affiliations:** 1 Department of Epidemiology, School of Public Health, University of Alabama at Birmingham, Birmingham, AL, USA; 2 Department of Biostatistics, School of Public Health, University of Alabama at Birmingham, Birmingham, AL, USA; 3 National Space Science and Technology Center, NASA Marshall Space Flight Center, Huntsville, AL, USA; 4 Division of Public Health Sciences, Wake Forest University School of Medicine, Winston-Salem, NC, USA

## Abstract

**Background:**

Evidence is mounting regarding the clinically significant effect of temperature on blood pressure.

**Methods:**

In this cross-sectional study the authors obtained minimum and maximum temperatures and their respective previous week variances at the geographic locations of the self-reported residences of 26,018 participants from a national cohort of blacks and whites, aged 45+. Linear regression of data from 20,623 participants was used in final multivariable models to determine if these temperature measures were associated with levels of systolic or diastolic blood pressure, and whether these relations were modified by stroke-risk region, race, education, income, sex hypertensive medication status, or age.

**Results:**

After adjustment for confounders, same-day maximum temperatures 20°F lower had significant associations with 1.4 mmHg (95% CI: 1.0, 1.9) higher systolic and 0.5 mmHg (95% CI: 0.3, 0.8) higher diastolic blood pressures. Same-day minimum temperatures 20°F lower had a significant association with 0.7 mmHg (95% CI: 0.3, 1.0) higher systolic blood pressures but no significant association with diastolic blood pressure differences. Maximum and minimum previous-week temperature variabilities showed significant but weak relationships with blood pressures. Parameter estimates showed effect modification of negligible magnitude.

**Conclusions:**

This study found significant associations between outdoor temperature and blood pressure levels, which remained after adjustment for various confounders including season. This relationship showed negligible effect modification.

## Introduction

Pathways contributing to the development of hypertension are complex, and blood pressure (BP) levels are affected by season [1,2]. There is growing evidence that outdoor temperature is a leading factor for seasonal fluctuations in blood pressure, resulting in higher blood pressures during the lower temperatures that occur in the winter and lower blood pressures during the warmer summer temperatures [3]. Exposure of skin to colder temperature results in an autonomic response that leads to vasoconstriction and directly to higher BP levels [4]. There is considerable geographic variation in both temperatures and the risk of stroke death, raising the possibility that variations in temperature may either synergize or ameliorate the underlying pattern of geographic disparities in stroke risk.

Temperatures may have differing effects on autonomic and cardiovascular systems of different racial and ethnic groups, introducing the possibility that temperature is a contributor to the higher cardiovascular mortality observed in African-Americans compared to whites [5-7]. Those with fewer years of education or income may be more exposed to outdoor temperatures since they more likely have poorer indoor temperature control and may have careers which require more time outdoors [8,9]. Antihypertensive therapy has been found to modify autonomic responses and hence autonomic tone, which potentially affects the presumed increase in peripheral vasomotor tone related to the colder temperatures in winter [10]. Previous studies indicate that outdoor temperature may have stronger effects on females, due to clothing choices or biological differences [11]. Older populations may be more susceptible to the effects of temperature on blood pressure, since many cardiovascular risk factors, such as arterial stiffening, worsen with age [3].

We examined the relationship between outdoor temperature and seasonality with blood pressure by linking data from a national longitudinal study with weather information available from satellite and ground-level assessments. In addition, we examined whether these relationships are modified by stroke-risk region, race, education, income, sex, hypertensive medication status, or age. To our knowledge, this study is the first large cohort study that includes a substantial representation of both black and white participants from the United States to explore these relationships performed.

## Materials and methods

### Participants

The assessment of the temperature-BP relationship was performed using participants from the REasons for Geographic And Racial Differences in Stroke (REGARDS) study. In brief, REGARDS is a longitudinal study with the goal of understanding racial and regional health disparities in stroke mortality and incidence [12]. The participants are aged 45 years and older and are sampled from the 48 conterminous United States. In the obtained sample, excluding those with unacceptable geocoding, 36% of the study participants were sampled from the "Stroke Belt", a high stroke mortality region consisting of the eight southeastern states of Arkansas, Louisiana, Tennessee, Mississippi, Alabama, Georgia, North Carolina, and South Carolina; 17% were sampled from the "Stroke Buckle", a region with even higher stroke mortality along the coastal plains of Georgia, North Carolina, and South Carolina; the remaining 47% were from the rest of the nation. Within each region the planned recruitment included half whites and half African-Americans (obtained sample, excluding unacceptably geocoded: 55% white, 45% African-American). Planned recruitment was half male and half female (obtained sample, excluding unacceptably geocoded: 45% male, 55% female). At baseline, a telephone interview was conducted which recorded the patient's medical history, personal history, demographic data, socioeconomic status, stroke-free status, depressive symptoms, and cognitive screening. An in-home exam was administered which recorded blood pressure, height, weight, venipuncture and urine collection, and electrocardiogram results. Participants were asked to remove outer clothing and shoes prior to physical measurements. All participants provided written informed consent, and the study was approved by the Institutional Review Board for Human Subjects at the University of Alabama at Birmingham, as well as all other participating institutions. Details on the study are available elsewhere [12].

The current residence from the original recruitment file plus updated information from the participant at the time of scheduling the in-home exam was used to establish each participant's address, which was then geocoded using SAS/GIS batch geocoding. Information obtained from SAS/GIS with 80% accuracy or greater was utilized in these analyses. The results from the SAS/GIS procedure were validated against a commercially available program http://www.geocode.com using the Haversine formula [13]. A mean difference of only 0.23 kilometers and a maximum difference of 0.95 kilometers were found between the two algorithms [14].

### Temperature assessment

Temperature values were prepared and provided by National Aeronautics and Space Administration's **(**NASA's) Marshall Space Flight Center. We obtained daily maximum and minimum temperatures for 2003 to 2006 from the North American Regional Reanalysis (NARR), a data product produced by the National Center for Environmental Prediction (NCEP), a division of the U.S. National Weather Service. The product includes data from satellites and ground observations and is composed of a 32 km resolution grid over North America. NARR daily maximum and minimum temperatures were matched to the latitude and longitude of each participant's geocoded residence. All temperature measures were indexed to the date that the in-home BP assessment was performed.

All temperature measurements were modeled as continuous variables. Maximum and minimum temperatures were characterized either as "same-day temperatures" taken solely from the day of the in-home visit or as "2-week temperatures" calculated as the average of the two weeks prior to the date of the in-home visit (inclusive).

We also examined temperature fluctuation and variability to determine whether the body's physiological adjustment and acclimatization, or possible behavioral changes (such as clothing choices, time spent outdoors) could have been involved in a participant's response to temperature [15,16]. Temperature fluctuations were calculated as the range of temperatures on the day of the in-home visit (same-day maximum minus same-day minimum), 2-day change (the difference between the same and previous days' maximum or minimum), or as the weekly variance of the daily maximum or minimum temperatures (week previous to the in-home visit, inclusive). Studentized residuals from linear models using weekly temperature variances to predict BP measures showed that the temperature variances should be log-transformed to achieve linearity and residual homogeneity. Thus, the natural log-transforms of week-long temperature variances were used to estimate weekly temperature fluctuations and for simplicity are hereafter referred to as the weekly "variabilities" of daily maximum or minimum temperatures.

### Blood pressure assessment

Systolic Blood Pressure (SBP) and Diastolic Blood Pressure (DBP) were determined from the REGARDS in-home visit. Blood pressure was measured by a trained technician using a standard protocol and regularly tested aneroid sphygmomanometer and was calculated as an average of two measurements taken after the participant was seated for five minutes.

### Participant selection

Data for the current analysis included 26,018 participants without previous stroke or TIA. Of these, 3,868 (14.9%) participants with unacceptable geocoding (less than 80% accuracy) were excluded from this analysis. An additional 11 (< 0.1%) participants were excluded because of an age under 45 years, and 245 (0.9%) because of implausible recorded blood pressure data (SBP not between 75 and 275 or DBP not between 50 and 150), reducing evaluable participants to 21,894. Missing values of any of the potential confounders (primarily glucose assessment missing 747 and BMI missing 279) eliminated an additional 1,271 (4.9%) during regression modeling. Although income data was missing for 2,652 participants, previous REGARDS methods were followed by creating a separate "refused" category in this variable so that fewer participants would be excluded during regression modeling.

### Statistical analyses

Linear regression models were used to assess the association between temperature and BP and to adjust for potential confounding by geographic region (stroke belt, stroke buckle, or non-stroke belt), population density defined by census tract (urban, mixed, and rural), individual income (less than $20,000, $20,000 to $34,999, $35,000 to $74,900, $75,000 and higher, or refused), community-based income status (percent of census tract under poverty), years of education (8^th ^grade or less), race (black or white), smoking (current, past, or never), alcohol use (never used or ever used), Body Mass Index (BMI) (underweight and normal, overweight, or obese), hyperlipidemia (cholesterol > 240), diabetes status (fasting glucose≥126, non-fasting glucose≥200, or self-reported diabetes medications), age in years, hypertensive medication status (currently taking vs. not currently taking), and astronomical season (fall, winter, summer, or spring) (10, 11, 12, 13, 14). The final multivariable models included all potential covariates. Parameter estimates with 95% confidence intervals (CIs) and p-values were calculated to measure the magnitude and strength of the associations between all predictors with DBP or SBP. In the final model we assessed interactions between temperature and race, region, education, income, sex, hypertensive medication status, and age. Lastly, likelihood ratio chi-squared and t-tests were used to assess differences between the 5,245 excluded and 20,773 included subjects in the final model.

## Results

2-week temperature averages and same-day temperatures both showed similar relationships with SBP and DBP in magnitude (all P < .0001); we used same-day measurements in model building since we knew participants were at or near their homes during this day. Daily temperature range and 2-day maximum and minimum temperature changes all showed non-significant associations with BPs (P > 0.05).

Table 1 shows higher SBPs and DBPs associated with lower maximum or minimum temperatures. Same-day maximum and minimum temperatures 20°F lower had significant associations with 1.5 or 1.1 mmHg higher SBPs, and 0.7 or 0.4 mmHg higher DBPs (Table 1). Temperature variabilities (defined as the log-transform of the weekly variances of daily temperatures) showed statistically significant relationships with SBP (Table 1). Maximum and minimum temperature variabilities a standard deviation higher were associated with approximately 0.5 mmHg higher SBPs (Table 1). Maximum and minimum temperature variabilities also showed statistically significant relationships with DBP, but small parameter estimates indicated associations of negligible magnitudes (Table 1).

**Table 1:** Univariate Relationships of Participant Characteristics with Blood Pressure (N = 21,894)

Characteristics	Distribution	Missing	SBP (mmHg)	DBP (mmHg)
	**N (%)**	**N**	**Parameter estimate (95% CI)**	**Parameter estimate (95% CI)**

**Outcome Variables**				

SBP, mmHg (mean, SD)	128.2 (16.7)	0	n/a	n/a

DBP, mmHg (mean, SD)	76.8 (9.6)	0	n/a	n/a

**Meteorological and Seasonal Variables**				

Same day maximum temp, lower by 20°F (mean, SD)	70.6 (16.8)	0	**1.5 (1.2, 2.0)**	**0.7 (0.5, 0.9)**

Same day minimum temp, lower by 20°F (mean, SD)	55.3 (16.8)	0	**1.1 (0.8, 1.4)**	**0.4 (0.3, 0.6)**

Maximum temp variability, by SD increase (mean, SD)	2.8 (1.4)	0	**0.5 (0.3, 0.6)**	**0.1 (0.0, 0.2)**

Minimum temp variability, by SD increase (mean, SD)	3.0 (1.4)	0	**0.6 (0.4, 0.7)**	**0.1 (0.0, 0.2)**

Season				

Summer	6184 (28%)		Reference	Reference

Fall	6041 (28%)	0	**1.4 (0.8, 2.0)**	**0.7 (0.3, 1.0)**

Winter	4853 (22%)		**2.3 (1.7, 2.9)**	**1.2 (0.8, 1.5)**

Spring	4816 (22%)		**2.0 (1.3, 2.6)**	**0.9 (0.5, 1.3)**

**Demographics**				

Age, by 10 years (mean, SD)	65.7 (9.3)	0	**2.9 (2.6, 3.1)**	**-1.3 (-1.1, -1.4)**

Male	9905 (45%)	3	**2.2 (1.7, 2.6)**	**1.1 (0.9, 1.4)**

Black Race	9527 (44%)	1	**5.4 (4.9, 5.8)**	**3.2 (3.0, 3.5)**

Region				

Stroke Buckle	3768 (17%)		Reference	Reference

Stroke Belt	7773 (36%)	0	**0.8 (0.1, 1.4)**	-0.1 (-0.5, 0.3)

Non-Belt	10325 (47%)		**1.5 (0.9, 2.1)**	**0.5 (0.2, 0.9)**

Population Density				

Urban	17678 (81%)		**1.7 (1.0, 2.5)**	**0.5 (0.1, 1.0)**

Mixed	2179 (10%)	0	0.5 (-0.5, 1.5)	0.2 (-0.4, 0.7)

Rural	2037 (9%)		Reference	Reference

**SES Factors**				

Education, 8^th ^grade or less	2849 (13%)	23	**5.5 (4.9, 6.2)**	**0.9 (0.5, 1.2)**

Income				

< $20 k	4122 (19%)		**8.0 (7.2, 8.7)**	**1.2 (0.7, 1.6)**

$20 k-$35 k	5367 (25%)	2652	**4.4 (3.7, 5.0)**	**0.8 (0.4, 1.1)**

$35 k-$75 k	6487 (30%)		**1.7 (1.0, 2.3)**	**0.4 (0.0, 0.8)**

>= $75 k	3266 (15%)		Reference	Reference

refused	2652 (12%)		**2.7 (1.9, 3.5)**	**1.1 (0.7, 1.6)**

Community poverty %, by 10% increase (mean, SD)	17.8 (12.5)	0	**1.6 (1.4, 1.7)**	**0.8 (0.6, 0.8)**

**Health Behaviors**				

Smoking Status				

Never	9670 (44%)		Reference	Reference

Current	3212 (15%)	88	**1.2 (0.5, 1.8)**	**0.6 (0.3, 1.0)**

Past	8924 (41%)		**1.1 (0.6, 1.6)**	-0.2 (-0.5, 0.1)

Alcohol use (never)	6486 (30%)	0	**1.2 (0.8, 1.7)**	-0.3 (-0.6, 0.0)

**Co-morbidities**				

BMI		279		

Underweight/Normal	5402 (25%)		Reference	Reference

Overweight	7972 (37%)		**3.4 (2.8, 3.9)**	**2.3 (2.0, 2.6)**

Obese	8241 (38%)		**6.9 (6.3, 7.4)**	**4.8 (4.5, 5.1)**

Hyperlipidemia	19254 (88%)	16	0.1 (-0.6, 0.8)	**1.5 (1.1, 1.9)**

Diabetes	4672 (22%)	747	**5.4 (4.8, 5.9)**	0.1 (-0.2, 0.5)

SBP = systolic blood pressure; DBP = diastolic blood pressure; SD = standard deviation; high temp = high temperatures; low temp = low temperatures; F = Fahrenheit

Parameter estimates in **bold **indicate values that are significant at α = 0.05.

SBP and DBP showed significant univariate relationships with season; mean SBP was approximately 2 mmHg higher and mean DBP was about 1 mmHg higher in the winter than the summer (Table 1). All other covariates (age, gender, race, region, population density, education, income, community poverty, smoking status, alcohol use, BMI, diabetes, and current hypertensive medication status) with the exception of dyslipidemia had significant relationships with SBP (Table 1). DBP was also significantly related to season and all other covariates, with the exceptions of alcohol use and diabetes status (Table 1).

Table 2 shows the "basic adjusted" multivariable models that added the demographic, socio-economic status, health behavior, and co-morbidity confounders to the regression models relating same-day temperatures to BP levels. Table 2 also shows the effect of adding weekly temperature variability and season to these basic adjusted models. There is no difference between the univariate and basic adjusted model parameter estimates for the relationships between same-day temperatures and BPs (Tables 1 and 2). The addition of temperature variabilities also does not change these parameter estimates, except for a small attenuation in the estimate for the relationship between daily minimum temperatures and SBP (Table 2). The addition of season to these models did not change the estimates of any of the relationships, except for nullifying the already weak relationship between same-day minimum temperatures and DBP, and a small attenuation of the relationship between weekly minimum temperature variability and SBP (Table 2).

**Table 2:** Multivariable Relationships of Temperatures with Blood Pressure (N = 20,773)

Temperature-BP models	Same-Day Maximum Temperatures (mmHg difference associated with 20°F lower)	Weekly Maximum Temperature Variability (mmHg difference associated with a SD higher variability)	Same-Day Minimum Temperatures (mmHg difference associated with 20°F lower)	Weekly Minimum Temperature Variability (mmHg difference associated with a SD higher variability)
	**Maximum temperature - SBP Model**		**Minimum temperature - SBP Model**	

Basic adjusted model *	1.5 (1.3, 1.8)	--	1.1 (0.8, 1.3)	--

Adjusted (with variability)**	1.4 (1.1, 1.6)	0.4 (0.1, 0.6)	0.7 (0.5, 1.0)	0.7 (0.4, 0.9)

**Adjusted (with variability and season)^#^**	1.4 (1.1, 1.8)	0.4 (0.1, 0.6)	0.7 (0.4, 1.1)	0.5 (0.2, 0.8)

	**Maximum temperature - DBP Model**		**Minimum temperature - DBP Model**	

Basic adjusted model *	0.7 (0.5, 0.8)	--	0.4 (0.3, 0.6)	--

Adjusted (with variability)**	0.7 (0.5, 0.9)	0.0 (-0.2, 0.1)	0.4 (0.2, 0.5)	0.1 (0.0, 0.3)

**Adjusted (with variability and season)^#^**	0.5 (0.3, 0.8)	-0.1 (-0.2, 0.1)	0.1 (-0.1, 0.4)	0.0 (-0.2, 0.1)

BP = blood pressure; SBP = systolic blood pressure; DBP = diastolic blood pressure; SD = Standard Deviation

* adjusted for sex, region, population density, income, community poverty, education, race, smoking, alcohol, Body Mass Index, hyperlipidemia, diabetes, and age

** adjusted for sex, region, population density, income, community poverty, education, race, smoking, alcohol, Body Mass Index, hyperlipidemia, diabetes, age, and weekly temperature variability

# adjusted for sex, region, population density, income, community poverty, education, race, smoking, alcohol, Body Mass Index, hyperlipidemia, diabetes, age, weekly temperature variability, and season

Parameter estimates in **bold **indicate values that are significant at α = 0.05.

**Figure 1:**
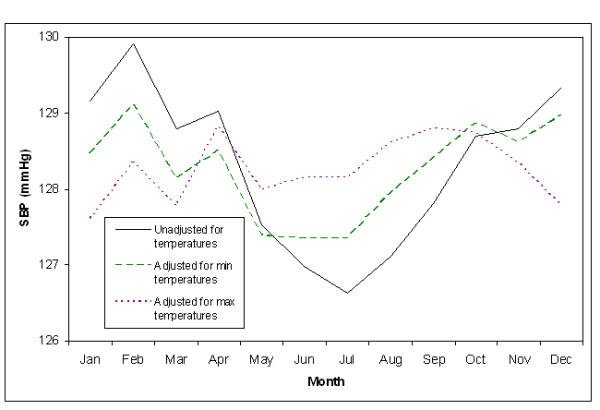
**Monthly SBP Adjusted for Demographics, SES Factors, Behavior, and Co-morbidities**.

**Figure 2:**
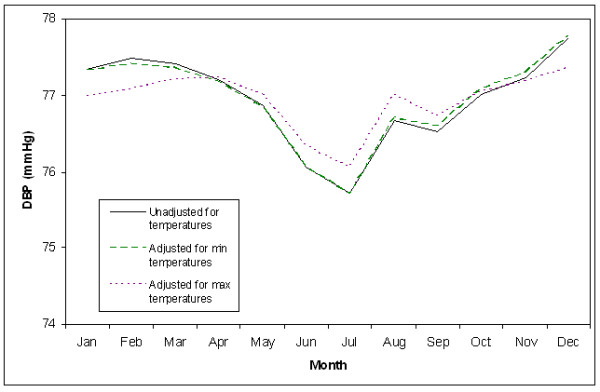
**Monthly DBP Adjusted for Demographics, SES Factors, Behavior, and Co-morbidities**.

In final multivariable models, season was significant in all models, except for the model assessing the relationship between maximum temperatures and SBP. Specifically, in the final model assessing the relationship between minimum temperatures and SBP, compared to summer, fall had 1.0 mmHg higher and winter had 1.2 mmHg higher SBPs (P = 0.001 and P = 0.002); spring was not significantly different (P = 0.39). In the final model assessing the relationship between minimum temperatures and DBP, compared to summer, fall had 0.7 mmHg, winter had 1.2 mmHg, and spring had 0.9 mmHg higher DBPs (all P < .0001). This is shown in Figures 1 and 2, which display monthly blood pressure averages adjusted for all covariates in the final models, aside from season. These figures also show that the seasonal differences in blood pressure are largely explained by temperature. Figure 1 shows that after adjustment for maximum temperatures and maximum temperature variability, this seasonal variation in SBP is highly attenuated and any seasonal pattern is difficult to discern. Adjustment for minimum temperatures and minimum temperature variability also appears to attenuate the seasonal variation in SBP, but to a lesser degree. In Figure 2 maximum temperatures and maximum temperature variability, but not minimum temperatures and minimum temperature variability appear to attenuate DBP.

**Figure 3:**
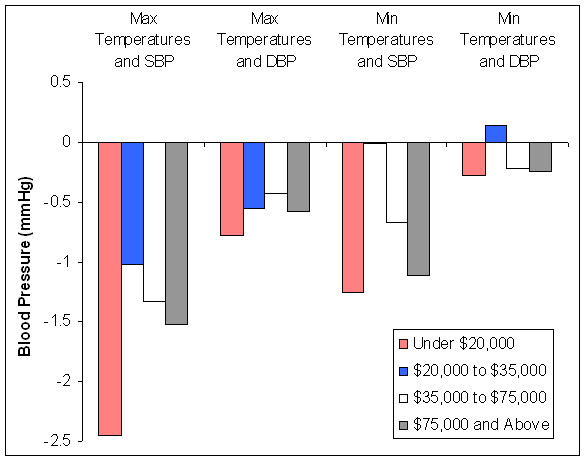
**Differences in Blood Pressures Associated with a 20°C Higher Same-Day Temperatures, by Individual Income**.

Chi-square tests on interaction variables indicated that income did not significantly modify the relationships between same-day temperatures and DBP (P_interaction _> 0.05), but did between same-day temperatures and SBP (P_max temps _= 0.02 and P_mintemps _= 0.03). Figure 3 shows the associations between same-day temperatures and blood pressures by income, but leaves out those who "refused" income information from the graph. The only income category with significantly different associations between same-day temperatures and BP was $20,000 to $35,000 (Figure 3). However, associations among the upper two income categories did not significantly differ from those making less than $20,000 (Figure 3).

The associations between same-day temperatures and blood pressure did not significantly differ by sex (P_interaction _> 0.05), with the exception that the association between high temperatures and DBP were stronger among females than males (P_interaction _= 0.001). However, a 20°F maximum temperature difference was associated with less than 1 mmHg DBP in both males and females (0.3 and 0.8 mmHg respectively).

Race, stroke-risk region, education, anti-hypertensive medication use, and age did not show effect modification on any of the relationships between same-day temperatures and BP (P_interaction _> 0.05). After accounting for any significant interactions, the associations between a standard deviation change in temperature variability and SBP or DBP still did not exceed 1 mmHg, so we did not display any of this data.

Table 3 shows that most of the variables had different distributions between participants in the final model and those who were excluded. The excluded participants were much more rural and less urban than the included.

**Table 3:** Final Covariates of Excluded and Modeled Participants

Characteristics	Missing	Excluded Participants	Participants in the Final Model	P-value
	**N**	**N (%)**	**N (%)**	

**Total**		5245 (20%)	20773 (80%)	

**Outcome Variables**				

SBP mmHg (SD)	0	127.8 (18.8)	128.2 (16.7)	0.20

DBP mmHg (SD)	0	77.1 (22.8)	76.8 (9.6)	0.43

**Meteorological Variables**				

Same day maximum temp, lower by 20°F (mean, SD)	0	70.4 (17.0)	70.6 (16.8)	0.52

Same day minimum temp, lower by 20°F (mean, SD)	0	55.2 (17.1)	55.2 (16.8)	0.76

Maximum temp variability, by SD increase (mean, SD)	0	2.0 (1.0)	2.0 (1.0)	0.008

Minimum temp variability, by SD increase (mean, SD)	0	2.2 (1.0)	2.1 (1.0)	<.0001

Season				

Summer		1450 (28%)	5872 (28%)	

Fall	0	1400 (27%)	5721 (28%)	0.003

Winter		1289 (25%)	4601 (22%)	

Spring		1106 (21%)	4579 (22%)	

**Demographics**				

Age, years (mean, SD)		65.3 (9.4)	65.7 (9.3)	0.004

Male	5	2251 (43%)	9481 (46%)	0.0005

Black Race	8	1926 (37%)	3311 (43%)	<.0001

Region				

Stroke Buckle		1195 (23%)	3605 (17%)	

Stroke Belt	0	2010 (38%)	7343 (35%)	<.0001

Non-Belt		2040 (39%)	9825 (47%)	

Population Density				

Urban		2916 (56%)	1952 (9%)	

Mixed	0	418 (8%)	2071 (10%)	<.0001

Rural		1911 (36%)	16750 (81%)	

**SES Factors**				

Education < = 8^th ^Grade	24	726 (14%)	2686 (13%)	0.06

Income				

< $20 k		1069 (20%)	3957 (19%)	

$20 k-$35 k	0	1318 (25%)	5112 (25%)	

$35 k-$75 k		1472 (28%)	6186 (30%)	0.0008

>= $75 k		721 (14%)	3135 (15%)	

refused		665 (13%)	2483 (12%)	

**Health Behaviors**				

Smoking Status				

Never		2376 (46%)	9170 (44%)	

Current	97	764 (15%)	3045 (15%)	0.01

Past		2008 (39%)	8558 (41%)	

Alcohol use (never)	0	1704 (32%)	6107 (29%)	<.0001

**Co-morbidities**				

BMI				

Underweight/Normal	362	1210 (25%)	5183 (25%)	

Overweight		1798 (37%)	7677 (40%)	0.92

Obese		1875 (38%)	7913 (38%)	

Hyperlipidemia	19	4085 (78%)	18848 (91%)	<.0001

Diabetes	895	980 (23%)	4565 (22%)	0.42

SBP = systolic blood pressure; DBP = diastolic blood pressure; SD = standard deviation; High Temp = high temperatures; Low Temp = low temperatures

P-values for categorical variables provided from a chi squared test statistic

P-Values provided from a pooled t-test statistic or Satterwaithe t-test calculated for each variable by inclusion status. The test was chosen depending on the results for a Folded f equality of variances test.

## Discussion

This study has the novel finding that the time of year, characterized by season, is secondary in importance to temperature's association with blood pressure. In this national sample of African-American and white participants aged 45 years and older, colder temperatures were associated with higher blood pressure measurements. These findings persisted regardless of using daily maximum or daily minimum temperatures, regardless of using same-day or the 2 week average temperatures as exposure measures, and also whether assessing SBP or DBP as the outcome. Temperature variability from the week previous to the blood pressure measurement had significant relationships with blood pressure, although these tended to be weak and somewhat inconsistent. Adjustment for confounders, including season, had little impact on these relationships. Relationships between temperature and blood pressure had negligible differences by stroke-risk region, race, education, income, sex, hypertensive medication status, or age. Daily range (day high minus day low) and 2-day change (previous day high minus current day high) were not found to be associated with BP.

This study adds to the body of literature showing that blood pressure varies by season, but demonstrates that this relationship is likely driven by temperature, rather than other factors that vary with the time of year, such as exercise, stress levels, mood, cognitive function, and various health behaviors and biological processes [17-24]. Furthermore, this study finds that the previous associations and estimates in earlier studies regarding this relationship were not likely inflated or due to Type I error as can be common with newly discovered associations [25]. Several studies have shown that BPs measured in the summer months are generally lower than BPs measured in the winter months; that colder climates are associated with higher BPs; and that residents in climates with greater seasonal temperature differences show greater BP fluctuations [1,11,18]. Other risk factors of hypertension and stroke such as BMI and cholesterol worsen in the winter months and higher levels of inflammatory biomarkers are associated with colder outdoor temperatures [2,26]. Clinical human and animal models have shown clear BP responses due to temperature changes [27]. Human and animal studies have demonstrated physiological mechanisms for this response, such as dehydration, arterial stiffness, and factors involved in sympathetic activation such as isoprenaline-induced relaxation of aortae and the rennin-angiotensin system [19,27-30]. Many studies report a seasonal pattern to stroke, which has a strong relationship with BP [31]. Stroke prone rats have been found to have exaggerated BP responses to cold exposure [32].

Our results indicated that the relationship of temperature with SBP or DBP differed only negligibly by stroke-risk region, race, education, income, sex, hypertensive medication status, or age. Given the large sample size of the REGARDS study, we feel that there was good statistical power to detect such differences if they did exist. Previous studies gave rise to question of whether environmental stresses have differing effects on black populations compared to white populations, but there were no racial differences in the relationships explored in our study [5,33-35]. Besides race, stroke-risk region was considered as an effect modifier. Since the Southeast has a particularly high incidence of stroke, it is of interest to determine if temperatures have differing effects on blood pressure in this region compared to others. However, our results gave the overall conclusion that there was no identifiable difference in the relationship by stroke-risk region. There may have been a difference in the relationship between temperature and BP among those making less than $20,000 a year as compared to those making more, but the results were not sufficiently clear to be definitive. This interaction might be explored in future studies. We did not find any differences in relationships by education. Sex and age have previously been shown to modify the relationship between temperature and blood pressure [3,11]. Our study did not find this to be the case.

This study was one of the few large population-based studies looking at associations of directly measured outdoor temperature and blood pressure [3,11]. Alpérovitch et al., 2009, a study that examined 8,801 subjects from 3 cities in different parts of France, found large blood pressure differences associated with longitudinal temperature differences [3]. A 15°C drop in temperature between baseline and follow-up was associated with a 2.3 mmHg SBP rise. A 15°C rise in temperature was associated with a 9.7 mmHg SBP drop. The difference between these two associations can be partially explained by the study's finding that even without any difference in temperature there was still an association with a 3.6 SBP drop, although it was not explained why this occurred. A panel study, Barnett et al., 2007, found similar associations between temperature drops and BP [11]. This study population consisted of over 115,000 subjects located in various European countries, as well as Canada, USA, New Zealand, and Australia. Barnett et al., 2007 did not differentiate between the changes in blood pressure associated with longitudinal temperatures rises vs. temperature falls as Alpérovitch et al., 2009 did. Their blood pressure changes were very similar to those associated with the temperature drops in Alpérovitch et al., 2009, but much smaller than those associated with temperature rises. Barnett et al., 2007 differed from Alpérovitch et al., 2009 by its study design (panel vs. cohort) and model choice, a hierarchical model with a season curve and temperature trend. It also accounted for indoor temperature's relationship with BP (0.31 mmHg rise per 1°C drop), which may have acted as a confounder in our study. The results from Barnett et al., 2007 were similar to ours, even though we were unable to account for indoor temperatures. While we attempted to partially account for indoor temperature differences using education and income, these were not strong effect modifiers

These two previous studies also differed by population ages. Alpérovitch et al., 2009 had an age range of 65 years and older, and in cross-sectional analyses found no interaction between age and temperature, which agrees with our study [3]. However, Alpérovitch et al., 2009 found an interaction between temperature and age in its longitudinal analyses. Barnett et al., 2007 had a younger population aged 35-64 years old. These two studies and other previous studies suggest the blood pressures of older populations may be more susceptible to longitudinal blood pressure changes due to drops or rises in temperature [2,7,20]. We did not have longitudinal data to compare to the results of the two previous studies and the lower bound of our age range was 45, as opposed to 35 in Barnett et al, 2007.

A limitation of these previous studies is that they did not account for racial or other demographic differences unique to U.S. populations and climates. REGARDS is representative of African-Americans and whites across the entire 48 conterminous United States, with regional and racial sampling biases. Cardiovascular risk factors and relationships differ by country and have been shown to be different between the US and both countries with both developed and emerging economies across the world [36]; this study shows the relationship as it applies to a US population sample and furthermore specifies in which racial groups the relationship was studied. In this way, we were able to determine that it is not likely that race acts as an effect modifier on the relationship between temperature and blood pressure.

Due to the high proportion of participants (21%) excluded for poor geocoding or missing covariate data, bias remains a possible limitation of this study. Table 3 shows that excluded participants significantly differed from those included in the final analysis. Differences occurred largely because rural participants were more likely to have poor geocoding scores, since rural route addresses frequently result in an inability to provide accurate geocoding of the residence. We do not know why the findings in this study would differ between urban and rural participants, and as such we suggest that this is not likely to be a substantial shortcoming of the study. Covariates with larger differences (over 3% differences in estimates) show our analyses had a disproportionate inclusion into the model of participants who were female, black, non stroke-belt residents, urban (vs. rural), and had high cholesterol (Table 3). These variables all have known relationships with blood pressure and would be the most likely causes of any bias, which might have resulted in underestimating or overestimating the relationship between temperature and blood pressure. Exposure misclassification exists as a possible source of bias for the study. This could happen if during the time period of an exposure measurement a participant spent a large amount of time in a climate different from that indicated by the outdoor temperatures linked to his or her residence. Examples of this would be if during a time when the temperatures were cold, the participant spent most of the time inside in a well-heated building or went on a vacation to somewhere warmer. This is a notable limitation, since room temperature, apart from the season and outside temperature has a direct effect on BP level [37]. Given that the same-day temperature measurements were taken on the same day as the in-home visit, the outdoor temperatures are likely to be valid for these measures. However, an ideal study would incorporate into the study the subjects' indoor temperatures and time spent indoors. Another source of bias arises from the fact that BP varies by time of day when recorded depending upon circadian rhythm. Ideally the study would have information regarding what time the BP measures were taken, and even more ideally would obtain a fuller picture of an individual's blood pressure by taking ambulatory measurements [7,38]. Another issue is regarding the cross-sectional nature of this study. Cross-sectional studies lack temporal evaluations between the relationships. There is more confidence in associations obtained from multiple measures on the same subjects to determine intra-participant BP variations. Another possible limitation involves the methods of our analysis. We chose to model season as 4 discrete categories, rather than using a spline or other continuous method of seasonal adjustment, thus there may be residual confounding. One final limitation is that there may be confounders for which we have not accounted. In particular, there may be other environmental variables that correlate with this relationship. It has been posited that other seasonal variables, such as sunlight, may have an effect on blood pressure. However, previous studies have found that atmospheric pressure (which can be used as a proxy for sunny weather), rainfall, and humidity were not related to blood pressure [20,39-41]. Our study had no measures of air pollution, which has been found to be related to blood pressure and other cardiovascular outcomes and was not accounted for in this study [26]. Taken together, these limitations would be addressed in future ideal studies by taking into account the previously mentioned concerns of indoor temperatures, times of day, and environmental factors, and the study would consist of repeated longitudinal participant measures showing temperature and blood pressure changes within single individuals.

Our study indicates that future studies looking at environmental impacts on blood pressure might not only use absolute temperature measurements, but also temperature variances. However, if the researcher has model simplification as a priority, our results indicate that this relationship might be best captured by using daily maximum temperatures as the exposure and SBP as the outcome. While it is possible these relationships differ by region, race, education, income, sex, hypertensive medication status, or age, our study provides evidence that the differences are likely not large.

## Abbreviations

BMI: Body Mass Index; BP: blood pressure; CI: confidence interval;DBP: Diastolic Blood Pressure; NARR: North American Regional Reanalysis; NASA: National Aeronautics and Space Administration; NCEP: National Center for Environmental Prediction; REGARDS: Reasons for Geographic And Racial Differences in Stroke; SBP: Systolic Blood Pressure;

## Competing interests

The authors declare that they have no competing interests.

## Authors' contributions

STK performed the analysis and wrote the manuscript. LAM provided statistical and GH provided epidemiological guidance and was involved in the generation of the hypothesis. WLC provided meteorological expertise and provided the NASA data. RJP provided epidemiological and medical expertise. All authors read and approved the final manuscript.
